# Effects of the addition of leucine on flavor and quality of sausage fermented by *Lactobacillus fermentum* YZU-06 and *Staphylococcus saprophyticus* CGMCC 3475

**DOI:** 10.3389/fmicb.2022.1118907

**Published:** 2023-02-02

**Authors:** Rui Liu, Yong Ma, Lei Chen, Chenyan Lu, Qingfeng Ge, Mangang Wu, Jun Xi, Hai Yu

**Affiliations:** ^1^School of Food Science and Engineering, Yangzhou University, Yangzhou, Jiangsu, China; ^2^Changshou Characteristic Meat Product Processing and Engineering Research Center of Jiangsu, Jiangsu Changshou Group Co., Ltd., Rugao, Jiangsu, China

**Keywords:** fermented sausage, leucine, Lactobacillus fermentum YZU-06, 3-methylbutanal, flavor

## Abstract

Methyl-branched aldehydes, especially 3-methylbutanal, have been reported to be perceived either as a malty or as a nutty/chocolate-like aroma and were considered an important flavor contributor in fermented meat products. Decomposition of leucine (Leu) by branched-chain amino acid transaminase (BACT) is a crucial step in the metabolism of Leu to 3-methylbutanal. This study was conducted to explore the effects of mixed-starter culture (*Lactobacillus fermentum* YZU-06 and *Staphylococcus saprophyticus* CGMCC 3475) and addition of Leu (0, 1, and 3 mM) on the flavor and quality of fermented sausages. The pH, water activity, texture profile analysis, color, counts of lactic acid bacteria (LAB) and staphylococci, peptide, and flavor compounds were detected during fermentation. The results showed that the starter culture group increased hardness, elasticity, the counts of LAB and staphylococci, peptide content, volatile flavor compounds, as well as the sensorial scores of sausage, while decreasing pH, a_*w*_, and L* and b* values compared with the non-inoculation group. The mixed starter of adding with 3 mM Leu enhanced the content of 3-methylbutanal and overall flavor of fermented sausages. It is applicable to directionally produce methyl-branched aldehydes and improve the overall quality of fermented sausage by the addition of Leu and using starter of *L. fermentum* YZU-06 and *S. saprophyticus* CGMCC 3475.

## 1. Introduction

Fermentation is an important process of meat preservation and the development of meat products, among which fermented sausage is favored by consumers worldwide. The texture and flavor of sausage are developed during fermentation involving many physical, microbiological, and biochemical changes. Flavor is one of the most important attributes of fermented sausage, consisting of esters, aldehydes, alcohols, ketones, acids, terpenes, aromatic, and compounds (Ammor et al., 2005; Baick and Kim, 2015). Branched-chain aldehydes are notably derived from precursors of branched-chain amino acids [BCAAs, i.e., leucine (Leu), isoleucine (Ile), and valine (Val)] that give the fermented sausage-rich aroma characteristics. Among them, the odor threshold is low for 3-methylbutanal, 0.06 ppm, followed by 2-methylbutanal (0.10 ppm), and 2-methylpropanal (0.10 ppm), which have been perceived as nutty and almond flavor in Hungarian Salami (Söllner and Schieberle, 2009). In addition, the presence of 3-methylbutanal, a degradation production of Leu, confers malt and chocolate aroma in fermented sausages (Smit et al., 2005). Olivares et al. (2009) determined the aroma-active compounds in fermented sausage at different processing stages and found that 3-methylbutanal was an important aroma contributor since the start of the ripening process. The relatively high concentrations of branched-chain aldehydes prominently contribute to the overall flavor of fermented sausage (Herranz et al., 2004).

Branched-chain amino acid transaminase (BCAT), the rate-limiting step of BCAA catabolism, is crucial to the biosynthesis of branched-chain aldehydes during fermentation (Afzal et al., 2012). The first step of synthetic branched-chain aldehydes is the formation of the corresponding α-ketoacids by the transamination reaction of BCAAs, which was catalyzed by BCAT. The α-ketoacid is subsequently decarboxylated and converted into its corresponding aldehydes (Afzal et al., 2012). The mutant of branched-chain aminotransferase gene (*bcaT*) in *Lactobacillus lactis* subsp. *cremoris* NCDO 763 would cause the subsequent decline in the production of intermediate keto acid (Yvon et al., 2000). Moreover, our previous study proved that *Lactobacillus fermentum* YZU-06 with high BCAT activity could be used for promoting BCAA metabolite synthesis in the myofibrillar protein model (Liu et al., 2022). From this perspective, the starter culture with high BCAT activity is able to contribute to the flavor of fermented meat products.

Flavor precursors are another essential factor in improving the aroma and quality of fermented meat products. Our previous study confirmed that the content of branched-chain aldehydes, alcohols, and acids, as well as texture properties, was improved by adding BCAAs in dry-cured fermented sausage (Wang et al., 2021). As an important precursor of flavor substances, protein is involved in the process of proteolysis to produce peptides and free amino acids (FAAs) (Yu et al., 2021). The application of protease can further promote proteolysis for better odor, flavor, texture, and general acceptability in dry fermented sausages (Bruna et al., 2002). In addition, lactic acid bacteria (LAB) have the ability to hydrolyze muscle proteins, contributing to flavor and/or aroma generation in products (Yu et al., 2020). Casaburi et al. (2008) reported that sarcoplasmic proteins were significantly degraded during sausage maturation by adding *Staphylococcus xylosus* and *L. curvatus*. Casaburi et al. (2006) found that *Staphylococcus saprophyticus* screened from fermented sausage was able to preferentially hydrolyze the N-terminal residue substrate, including leucine. The proteolytic ability of *S. saprophyticus* CGMCC 3475 has been confirmed in our previous work (data not shown). In this study, *S. saprophyticus* CGMCC 3475 was used to hydrolyze proteins and intended to release more aroma precursors (peptides and FAAs) for the flavor improvement of fermented sausage. Meanwhile, *L. fermentum* YZU-06 with both proteolytic and BCAT activity presented more BCAA metabolites of 3-methylbutanal and 2-methylbutanal than that of *L. plantarum* CGMCC18217 in myofibrillar protein model in our previous work (Liu et al., 2022). However, it is not clear whether the characteristic 3-methylbutanal can be directionally produced by adding exogenous Leu and *L. fermentum* YZU-06 in meat products. Therefore, the aim of this study was to evaluate the effects of *L. fermentum* YZU-06 in combination with *S. saprophyticus* CGMCC 3475 and the addition of Leu on the flavor and quality of sausage during the fermentation for the flavor development of a fermented product.

## 2. Materials and methods

### 2.1. Material preparation

*L. fermentum* YZU-06 and *S. saprophyticus* CGMCC 3475 were isolated from Jinhua ham and maintained at −80^°^C in de Man-Rogosa-Sharpe (MRS) medium containing 25% (v/v) glycerol. The strain of 1% (v/v) inoculum was activated twice and grown in 10 ml MRS broth at 37^°^C for 18 h before use. The bacterial cells were harvested by centrifugation at 6,000 × *g* for 10 min at 4^°^C. The pellet was washed three times with 0.1 M phosphate-buffered saline (PBS) solution (pH 7.0) and resuspended in PBS solution. Edible-grade Leu was purchased from Shanghong Biotech, Zhengzhou, China. Pork muscle and back fat were obtained from the carcasses aged for 24 h at 4^°^C in a commercial slaughter plant (Sushi Meat Co., Ltd., Huai’an, China). Glucose, salt, monosodium glutamate, spice powder, ginger powder, Daqu liquor, and distilled water were purchased from the Auchan’s Market (Yangzhou, China).

### 2.2. Preparation of fermented sausages

The fermented sausages were manufactured in accordance with Ge et al. (2019) with some modifications. Lean pork (35 kg) and pork fat (15 kg) were minced and mixed with the following ingredients: glucose (7%), salt (3%), monosodium glutamate (0.2%), spice powder (0.1%), ginger powder (0.15%), Daqu liquor (2%), and distilled water (10%). The mixture was divided into the following nine groups. The inoculation density of the single bacteria starter (*L. fermentum* YZU-06) or mixed-starter culture (*L. fermentum* YZU-06 and *S. saprophyticus* CGMCC 3475 at a 2:1 ratio) was 10^7^ CFU/g in the sausages. The first group without strains and Leu was assigned to the CK group. The groups added with 1 and 3 mM Leu were assigned to the CK-1 and CK-3 groups, respectively. The group fermented with *L. fermentum* YZU-06 was assigned to the G group. *L. fermentum* YZU-06 and Leu (1 and 3 mM) were added, designating as the G-1 and G-3 groups. The group inoculated with mixed-starter culture was assigned to the GQ group. The mixed-starter culture with different concentrations of Leu, including 1 and 3 mM, was added and named the GQ-1 group and GQ-3 group, respectively. Mixtures were filled in natural casings of 4 cm in diameter and 10 cm in length. Three independent batches of fermented sausages were prepared, and three sausages were performed in each batch. Sausages were placed in a fermentation room. In the first stage of fermentation, relative humidity (RH) and temperature were set to 95% and 30^°^C ± 0.5^°^C for 1 day. The temperature was adjusted to 16^°^C ± 0.5^°^C, and RH was successively decreased to 90, 87, and 85%. The duration for each RH was 2 days. The fermentation temperature in the third stage was 12^°^C ± 0.5^°^C, and RH was set at 85, 80, and 75%, and each RH condition was fermented for 7 days. The total ripening period was 28 days. Samples were taken from each group on days 1, 7, 14, 21, and 28 of fermentation.

### 2.3. pH value measurement

A digital pH value meter (Testo 205, Testo AG, Germany) was used to measure the pH value of fermented sausages after the casings were removed. Standard buffers of pH 4.0, 7.0, and 10.0 [Thermo Fisher Scientific (China) Co., Ltd., Shanghai, China] were used to calibrate the pH meter. The sausage samples (10 g) were homogenized with 50 ml distilled water for 10 s twice at 8,000 rpm on ice, with a 15 s interval between bursts. The pH was recorded and averaged from triplicate measurements.

### 2.4. Determination of water activity (a_*w*_)

The a_*w*_ was determined using the a_*w*_ meter (Novasina AG, Novasina, Switzerland). Sausages (3 g) were minced and placed in the measuring chamber for 10 min at 24^°^C. The a_*w*_ value was recorded after the measurement was completed.

### 2.5. Texture profile analysis (TPA)

The TPA of fermented sausages was detected and referred to the method of Ge et al. (2019) with slight modifications. Fermented sausages were cut into cubes in the shape of 1 cm × 1 cm × 1 cm and then measured by the texture analyzer (TA-XT. plus, Stable Micro Systems, Godalming Surrey, UK), which was equipped with a stainless-steel cylinder probe (P/36 R, Stable Micro Systems, Godalming Surrey, UK). The sausage sample was compressed to 50% of its original height with a pretest speed of 2 mm/min, a test speed of 2 mm/min, and a post-test speed of 1 mm/min. Hardness (N), adhesion (mJ), cohesion (ratio), elasticity (mm), and chewiness (mJ) were recorded. The TPA attributes of each sausage sample were averaged from triplicate measurements.

### 2.6. Determination of color of fermented sausages

The color was determined using a colorimeter (Chroma Meter CR-400, Konica Minolta, Japan) and calibrated with a white ceramic tile. The instrumental color parameters, including L*, a*, and b* values, of each sample were measured in triplicate from different sites.

### 2.7. Microbiological analysis

The microbiological analysis was performed according to Kaban and Kaya (2009) with slight modifications. A 10-g sausage was cut using a sterile knife and then transferred into a sterile plastic bag. The meat sample was homogenized with 90 ml sterile water using a homogenizer (80 microBiomaster, Seward, Britain) for 120 s with a 10 s interval between bursts. The serial 10-fold dilutions were prepared in sterile physiological water (0.85% NaCl). Appropriate dilution samples (0.1 ml) were poured or spread in duplicate on different growth media. LAB was enumerated on MRS agar (Hope Bio-Technology Co., Ltd., Qingdao, China) after 48 h at 30^°^C. *Staphylococcus* was determined on mannitol salt agar (Hope Bio-Technology Co., Ltd., Qingdao, China) after the incubation was carried out at 30^°^C for 48 h.

### 2.8. Peptide analysis

Peptides were determined according to the methodology described by Nie et al. (2014). The 3 g fermented sausage sample was homogenized with 27 ml of 15% TCA solution (w/v) using a homogenizer (T 18 digital, IKA group, Staufen, Germany) at 3,000 rpm for 60 s, followed by centrifugation at 12,000 × *g* for 10 min at 4^°^C. The content of soluble peptides in the supernatant was determined using a Stable Lowry Protein Assay Kit (C504051-1000, Shanghai Shenggong Co., Ltd., Shanghai, China) and expressed as μg/g sample.

### 2.9. Determination of volatile compounds

Volatile components were determined as described by Montanari et al. (2016) with some modifications. For each analysis, a 10 g sausage sample was minced and weighed into a 40 ml headspace vial, and methyl caprylate (0.68 mg/ml) was used as the internal standard solution by adding 20 μl into each headspace vial. A solid-phase microextraction needle covered with 75 μm carboxen/polydimethylsiloxane (CAR/PDMS Stable Flex) (Supelco, Steinheim, Germany) was exposed headspace of the sample for 45 min in the vial with 60^°^C water bath. After the adsorption of the volatile compounds, the fiber was injected and kept for 5 min in the inlet (230^°^C, splitless mode) of a gas-chromatograph-mass spectrometry (Trace ISQ, Thermo Scientific, Waltham, USA). The volatile compounds were separated using a 30 m × 0.25 mm × 0.2 μm capillary column (DB-WAX, Agilent, USA). The carrier gas was nitrogen (grade N_2_ > 99.999%) at a constant flow rate of 3.0 ml/min. The temperature of the detector was maintained at 280^°^C. The oven temperature was initially kept at 40^°^C for 1 min, increased by 5^°^C/min up to 100^°^C for 8 min, and then 8^°^C/min up to 240^°^C for 5 min. The ionization mode was EI^+^, and the electron energy was 70 eV. The scan time was 0.2 s, and the detector voltage was 500 V. The volatile compounds with similarities greater than 80% were shown by comparing the mass spectral data of the samples with those retrieved from digital libraries. The content of the aroma compound was expressed as relative content based on the internal standard.

### 2.10. Sensory evaluation

The sensory evaluation of fermented sausage was conducted with the method described by Valencia et al. (2006). There were 12 panelists (6 men and 6 women), aged between 20 and 35 years, majoring in Food Science at the School of Food Science and Engineering of Yangzhou University, Yangzhou, China. The panelist training, sensory room, and procedures of sensory evaluation were followed by the Chinese standard GB/T 22210-2008 (Criterion for sensory evaluation of meat and meat products). The sensory panelists received the training according to the nine-point scoring criteria (Table 1). The panelists were set in individual compartments, and all forms of communication were not allowed during the evaluation. Smoking, drinking, and eating were not allowed for at least 1 h before sensory analysis. The sample (0.3 cm thick) of each fermented sausage at room temperature was placed on a white ceramic plate labeled with a three-digit random code. Distilled water was provided to the panelists to rinse their mouths to avoid cross-interference. All the data were collected and used for statistical analysis.

**TABLE 1:** The definition of sensory quality attributes for fermented sausage.

Scoring items	Scoring criteria	Score
Color (9)	The cut meat filling is shiny, the muscle is rosy red, and the surface is bright	7–9
	The cut surface is glossy, the fat is yellow, and the muscle is dark	4–6
	The meat filling is dull and the muscle is gray	1–3
Flavor (9)	It has the characteristic aroma of fermented sausage	7–9
	The aroma of fermented sausage is weak	4–6
	Poor aroma and peculiar smell	1–3
Texture (9)	The cut meat filling is tight, the lean meat is closely combined with the fat meat, and the boundary is clear	7–9
	The meat filling on the cut surface is loose, and the combination of lean meat and fat meat is not tight	4–6
	The section is completely loose and the center is softened	1–3
Taste (9)	Salty and sweet, pure sour taste, pleasant aftertaste	7–9
	The taste is not pure and there is less aftertaste	4–6
	Strong sour taste and unpleasant aftertaste	1–3

### 2.11. Statistical analysis

The data obtained from the experiment were analyzed using SAS V8 software (SAS Institute Inc., Cary, NC, USA). Physical, microbial, and quality characteristic data were evaluated using a mixed-model ANOVA using Leu concentration (0, 1, and 3 mM), starter treatments (non-starter, single-strain starter, and mixed-strain starter), and fermentation time that were set as the main effect factors and batches as the random factor. For the sensory evaluation, the random effects included sausage and sensory panel (session number, tasting order, and panelist number). The mean standard error of each treatment was used to describe the least significant difference of means after the data were subjected to the Bonferroni test (*P* < 0.05). A principal component analysis was performed using the statistical software SIMCA 14.1 (MKS Instruments AB Inc., Malmo, Sweden).

## 3. Results and discussion

### 3.1. pH and a_*w*_ changes

**FIGURE 1:**
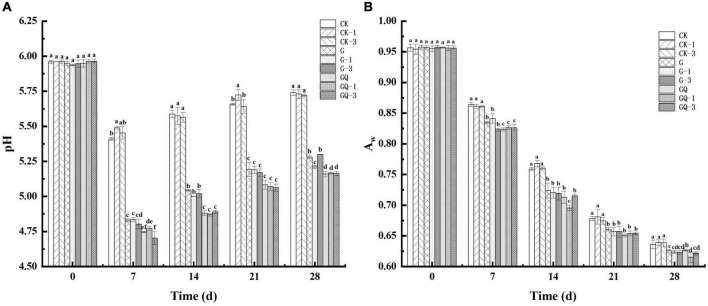
The pH values **(A)** and a_w_ **(B)** of fermented sausages among treatments during the fermentation for 0, 7, 14, 24, and 28 days. Different lowercase letters (a–f) indicate a significant difference between different treatment groups at the same fermentation time (*P* < 0.05). The group without starter culture and leucine was assigned to the CK group. The groups added with 1 and 3 mM of leucine without starter culture were assigned to the CK-1 and CK-3 groups, respectively. The group with *Lactobacillus fermentum* YZU-06 was assigned to the G group. The groups with *Lactobacillus fermentum* YZU-06 and different concentrations of leucine (1 and 3 mM) were named G-1 and G-3 groups, respectively. The fermented sausage with the mixed-starter cultures of *Lactobacillus fermentum* YZU-06 and *Staphylococcus saprophyticus* CGMCC 3475 was assigned to the GQ group. The groups with the mixed-starter cultures and different concentrations of leucine (1 and 3 mM) were named GQ-1 and GQ-3 groups, respectively.

The change in pH values during sausage fermentation is shown in Figure 1A. The initial pH value of all treatment samples was approximately 5.94, showing no significant difference among treatments (*P* > 0.05). A declining trend in pH values was observed in the first 7 days and then increased during the later fermentation, which may be due to the buffering effect of the peptides, and amino acids from proteolytic degradation and the organic acids produced by LAB during fermentation (Essid and Hassouna, 2013). The pH value of fermented sausages in the GQ, GQ-1, and GQ-3 groups was lower than that of the groups with and without *L. fermentum* YZU-06 inoculation during the fermentation. By the end of fermentation, the lowest pH values (a mean of pH 5.16) were found in the mixed-starter groups, which was significantly lower than those of other groups (*P* < 0.05). This may be due to the combined effects of the acid produced by *Lactobacillus* and *Staphylococcus* (Sun et al., 2016), which may contribute to the inhibition of the growth of spoilage microorganisms and prolonged shelf life of fermented sausage production (Adams and Nicolaides, 1997).

Figure 1B shows a decreasing trend in a_*w*_ of sausage through the entire fermentation process. The high a_*w*_ values (0.96) of the samples were detected at the beginning of the processing and then a_*w*_ decreased gradually to the lower values ranging from 0.61 and 0.64 at the end of fermentation. The imbalance in humidity between the inside and outside of the sausages causes moisture loss (Olivares et al., 2010). Moreover, the denaturation of proteins as a result of the drop in pH during fermentation and the degradation of protein caused by microorganisms involved in fermentation likely decreased the water-holding capacity (Seleshe and Kang, 2021). The starter and fermentation time was shown to have a cross-impact on a_*w*_ value. The a_*w*_ of the inoculated group was significantly lower than that of the control groups during incubation (*P* < 0.05), which was consistent with the finding that the lowest a_*w*_ value in the dry fermented sausage was fermented by *L. plantarum* GM77 and *S. xylosus* GM92 (Kaban and Kaya, 2009). The results suggested that the inoculation treatments promoted the reduction of a_*w*_, while the addition of Leu had a minimal effect on the a_*w*_ decline in sausage during fermentation.

### 3.2. Texture properties

**FIGURE 2:**
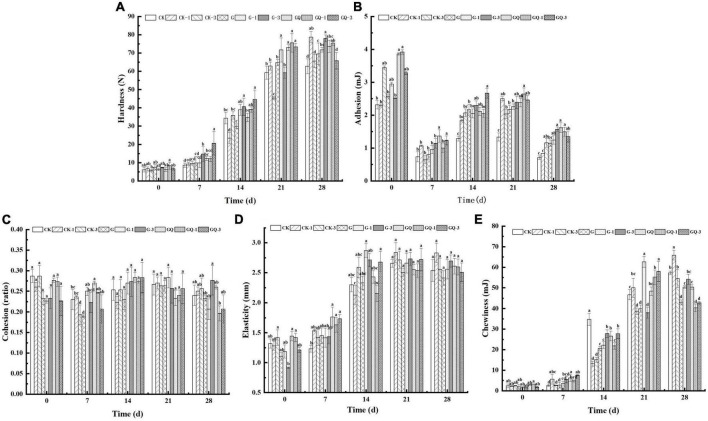
Changes in the texture of sausages, including hardness **(A)**, adhesion **(B)**, cohesion **(C)**, elasticity **(D)**, and chewiness **(E)**, among treatments during the fermentation for 0, 7, 14, 24, and 28 days. Different lowercase letters (a–d) indicate a significant difference between different treatment groups at the same fermentation time (*P* < 0.05). The group without starter culture and leucine was assigned to the CK group. The groups added with 1 and 3 mM of leucine without starter culture were assigned to the CK-1 and CK-3 groups, respectively. The group with *Lactobacillus fermentum* YZU-06 was assigned to the G group. The groups with *Lactobacillus fermentum* YZU-06 and different concentrations of leucine (1 and 3 mM) were named G-1 and G-3 groups, respectively. The fermented sausage with the mixed-starter cultures of *Lactobacillus fermentum* YZU-06 and *Staphylococcus saprophyticus* CGMCC 3475 was assigned to the GQ group. The groups with the mixed-starter cultures and different concentrations of leucine (1 and 3 mM) were named GQ-1 and GQ-3 groups, respectively.

As shown in Figure 2A, the hardness of the sausage increased significantly during 28 days of fermentation (*P* < 0.05). At the end of fermentation, the hardness of the inoculated groups was significantly higher than that of the control groups (*P* < 0.05), except for the CK-1 group. These results are in agreement with those found by Lorenzo and Franco (2012), who reported that the hardness was negatively related to a_*w*_ value in dry-cured foal sausages. On the contrary, the adhesion of sausages decreased at 28 days compared with the beginning of fermentation, and the adhesion of the inoculation groups was significantly higher than those of the CK and CK-1 groups (*P* < 0.05, Figure 2B). Figure 2C shows that no significant differences were observed in cohesion among treatment groups at 28 days (*P* > 0.05), and the addition of starter and Leu, as well as fermentation time, had no significant effects on cohesion (*P* > 0.05). Elasticity and chewiness increased with the fermentation time (Figures 2D, E). The starters are responsible for the fermentation of carbohydrates added to the mixture, leading to a decrease in the pH value. The acidification below the isoelectric point of muscle protein affects protein coagulation, which is responsible for the hardness and chewiness of the final product (Essid and Hassouna, 2013). The results of texture are consistent with the findings of Lorenzo and Franco (2012) that the change in TPA is attributed to the water loss in sausage during the maturing period.

### 3.3. Color changes

**FIGURE 3:**
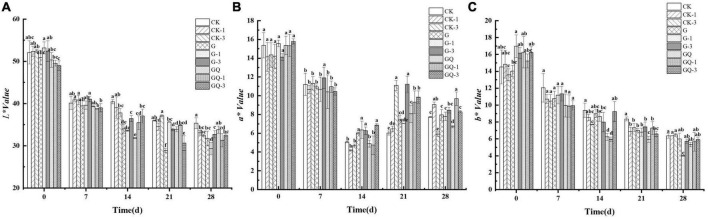
Changes in the color attributes of L* **(A)**, a* **(B)**, and b* **(C)** in sausages among treatments during the fermentation for 0, 7, 14, 24, and 28 days. Different lowercase letters (a–f) indicate a significant difference between different treatment groups at the same fermentation time (*P* < 0.05). The group without starter culture and leucine was assigned to the CK group. The groups added with 1 and 3 mM of leucine without starter culture were assigned to the CK-1 and CK-3 groups, respectively. The group with *Lactobacillus fermentum* YZU-06 was assigned to the G group. The groups with *Lactobacillus fermentum* YZU-06 and different concentrations of leucine (1 and 3 mM) were named G-1 and G-3 groups, respectively. The fermented sausage with the mixed-starter cultures of *Lactobacillus fermentum* YZU-06 and *Staphylococcus saprophyticus* CGMCC 3475 was assigned to the GQ group. The groups with the mixed-starter cultures and different concentrations of leucine (1 and 3 mM) were named GQ-1 and GQ-3 groups, respectively.

The changes in L*, a*, and b* values of fermented sausages are shown in Figure 3. All samples exhibited the typical color characteristics of fermented sausages. The L* value decreased from 51.11 to 32.01 in sausage during 28 days of fermentation (*P* < 0.05). At the end of fermentation, the L* values of the G-1 and GQ-1 groups were 29.33 and 31.32, respectively, which were significantly lower than that of the other groups (*P* < 0.05). The average a* value of the incubated samples decreased from 15.08 to 5.86 during 14 days of ripening and then increased to 8.15 at the end of the process. The b* value in all treatment groups decreased from 15.11 to 5.76 during the ripening. The b* value of the inoculated G-1 group was significantly lower than those of the control groups during 21–28 days of fermentation (*P* < 0.05). Color plays an important role in consumers’ sensory perception of fermented sausage. Casaburi et al. (2007) found that the L* value of sausages decreased throughout the entire fermentation period. Likewise, the decrease in L* value of Suck sausage within 15 days of maturation led to a decrease in gloss, which was consistent with the report by Kayaardı and Gök (2004). The decrease in a* value could be the denaturation of nitroso-erythropoietin due to the production of lactic acid. This result was in agreement with the findings of Ge et al. (2019), who found that the change in a* value in the *L. plantarum* NJAU-01 group was in the same trend as this study. The Spanish dry-cured sausages showed a decreasing trend in b* value during the maturation stages (Pérez-Alvarez et al., 1999). The decrease in b* value was probably due to the consumption of oxygen by microorganisms, resulting in a decrease in oxygenated myoglobin, which was an important component of the yellow coloration.

### 3.4. Changes in the count of LAB and staphylococci

**FIGURE 4:**
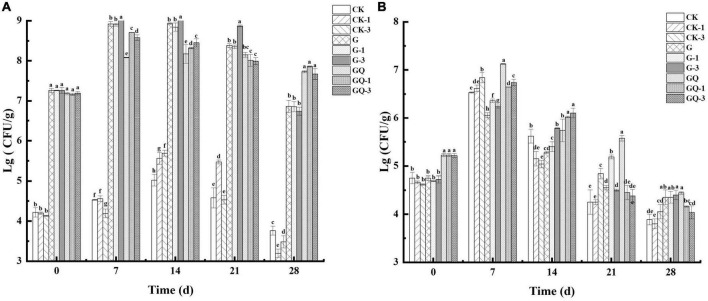
Changes in viable counts of LAB **(A)** and *staphylococcus* **(B)** in sausages among treatments during the fermentation for 0, 7, 14, 24, and 28 days. Different lowercase letters (a–h) indicate a significant difference between different treatment groups at the same fermentation time (*P* < 0.05). The group without starter culture and leucine was assigned to the CK group. The groups added with 1 and 3 mM of leucine without starter culture were assigned to the CK-1 and CK-3 groups, respectively. The group with *Lactobacillus fermentum* YZU-06 was assigned to the G group. The groups with *Lactobacillus fermentum* YZU-06 and different concentrations of leucine (1 and 3 mM) were named G-1 and G-3 groups, respectively. The fermented sausage with the mixed-starter cultures of *Lactobacillus fermentum* YZU-06 and *Staphylococcus saprophyticus* CGMCC 3475 was assigned to the GQ group. The groups with the mixed-starter cultures and different concentrations of leucine (1 and 3 mM) were named GQ-1 and GQ-3 groups, respectively.

The change in LAB counts in the sausages during fermentation is shown in Figure 4A. The addition of Leu had no significant effects on the initial LAB counts (*P* > 0.05). At the initial fermentation stage, the counts of MRS solid media in the sausage groups inoculated with LAB were about 7.17–7.27 lg CFU/g, and the counts in the control groups were 4.13–4.22 lg CFU/g. The inoculated groups with starter cultures reached the highest level of LAB (8–9 lg CFU/g) at 7 or 14 days of fermentation. After 7 days of fermentation, the counts of LAB in the GQ, GQ-1, and GQ-3 groups were lower than that of the G, G-1, and G-3 groups. However, at the end of the fermentation, the counts of LAB in the GQ, GQ-1, and GQ-3 groups were significantly higher than that in the G, G-1, and G-3 groups and the control groups (*P* < 0.05). This indicated that *L. fermentum* YZU-06 and *S. saprophyticus* CGMCC 3475 were well adapted to the environment and propagated rapidly during sausage fermentation. The addition of a starter inhibited the growth of undesirable bacteria, and the mixed starter promoted better growth of LAB (Essid and Hassouna, 2013). The low pH and a_*w*_ values may contribute to the decrease in the count of LAB in the fermented sausage at the end of ripening (Samelis et al., 1998).

Figure 4B shows the changes in the counts of staphylococci in the sausages during fermentation. The initial counts of staphylococci were 5.21–5.23 lg CFU/g in the sausages of GQ, GQ-1, and GQ-3 groups and 4.61–5.06 lg CFU/g in the sausages of LAB inoculated and control groups, respectively. The counts of staphylococci increased to 6.64–7.13 lg CFU/g, 5.62–6.36 lg CFU/g, and 6.53–6.84 lg CFU/g in inoculated staphylococci, inoculated LAB, and control groups at 7 days of fermentation, respectively. After the completion of the fermentation, the counts of staphylococci decreased to 3.80–4.45 lg CFU/g. The reduction in staphylococci during the last 2 weeks of maturation in sausages indicated that staphylococci were less competitive and inhibited by the intensive growth of LAB and a lower pH value (Casaburi et al., 2008).

### 3.5. Peptide content

**FIGURE 5:**
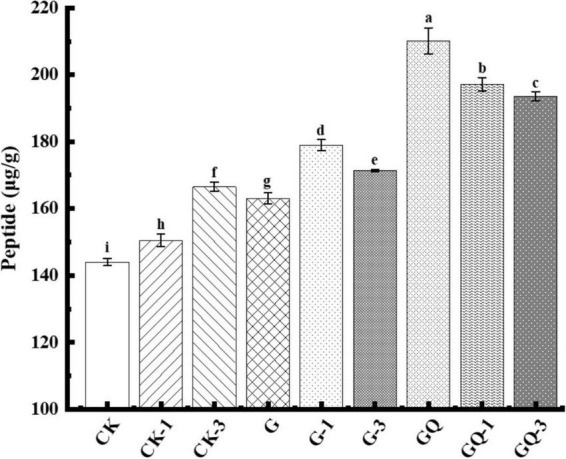
Peptide content of sausage among treatments at the end of fermentation (28 days). Different lowercase letters (a–i) indicate a significant difference between different treatment groups (*P* < 0.05). The group without starter culture and leucine was assigned to the CK group. The groups added with 1 and 3 mM of leucine without starter culture were assigned to the CK-1 and CK-3 groups, respectively. The group with *Lactobacillus fermentum* YZU-06 was assigned as the G group. The groups with *Lactobacillus fermentum* YZU-06 and different concentrations of leucine (1 and 3 mM) were named G-1 and G-3 groups, respectively. The fermented sausage with the mixed-starter cultures of *Lactobacillus fermentum* YZU-06 and *Staphylococcus saprophyticus* CGMCC 3475 was assigned to the GQ group. The groups with the mixed-starter cultures and different concentrations of leucine (1 and 3 mM) were named GQ-1 and GQ-3 groups, respectively.

The content of soluble peptides derived from the fermented sausages at the end of fermentation is shown in Figure 5. The peptide contents of GQ, GQ-1, and GQ-3 groups with the mixed starter and supplemented with Leu were significantly higher than the control groups CK, CK-1, and CK-3 (*P* < 0.05). The increase in total peptides indicated protease activity, mainly deriving from the action of microorganisms and endogenous muscle enzymes (Visessanguan et al., 2004). The higher total peptide content of the mixed inoculated group indicated a higher degree of protein degradation, which also showed that the higher content of the substance can be used as the precursor of flavor compounds. These peptides can be further decomposed by enzymes to produce some low-molecular-weight compounds, including amino acids, aldehydes, organic acids, and ammonia, which are potential flavor volatile (Díaz et al., 1993). For example, 3-methylbutanal, as a metabolite of BCAAs, can interact with different sulfur compounds derived from methionine and cysteine to form bacon flavor and improve sausage flavor (Riebroy et al., 2008). In addition, the rate of protein hydrolysis usually depends on many factors, including the nature of the meat microbiota and processing conditions. As reported by Visessanguan et al. (2004), the low pH value stimulated the hydrolysis of myofibrillar protein to form FAAs.

### 3.6. Volatile compounds

**FIGURE 6:**
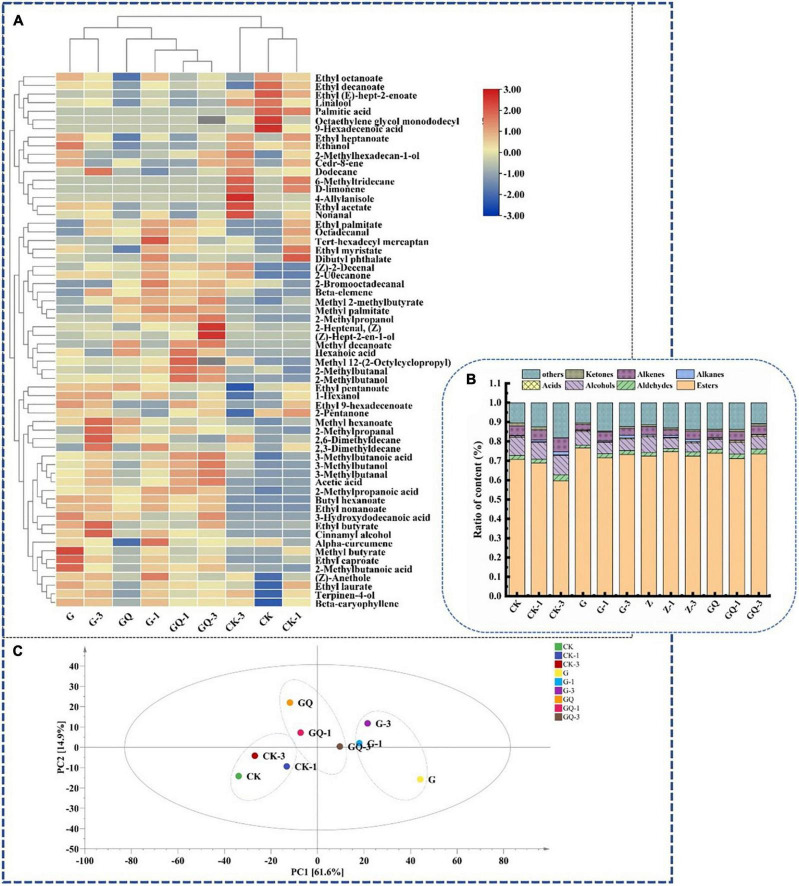
The flavor profiles of fermented sausages, including heatmap **(A)**, composition of sausage flavors in the different groups **(B)**, and principal component analysis of flavor substances among treatments **(C)** at the end of fermentation (28 days). The group without starter culture and leucine was assigned to the CK group. The groups added with 1 and 3 mM of leucine without starter culture were assigned to the CK-1 and CK-3 groups, respectively. The group with *Lactobacillus fermentum* YZU-06 was assigned to the G group. The groups with *Lactobacillus fermentum* YZU-06 and different concentrations of leucine (1 and 3 mM) were named G-1 and G-3 groups, respectively. The fermented sausage with the mixed-starter cultures of *Lactobacillus fermentum* YZU-06 and *Staphylococcus saprophyticus* CGMCC 3475 was assigned to the GQ group. The groups with the mixed-starter cultures and different concentrations of leucine (1 and 3 mM) were named GQ-1 and GQ-3 groups, respectively.

The identification of volatile components in fermented sausages at 28 days is shown in Figure 6. As shown in Figures 6A, total of 62 volatile compounds were determined and grouped into seven classes as shown in Supplementary Table 1, including esters (22), alcohols (11), aldehydes (8), acids (8), alkanes (4), olefins (5), ketones (2), and other compounds (2). Among them, 32, 47, and 43 compounds were detected in CK, CK-1, and CK-3 groups, respectively, while more than 49 compounds were detected in the inoculation group. The relative content of flavor substances in each treatment group is shown in Figure 6B. The main volatile flavor substances in fermented sausage were esters, aldehydes, alcohols, acids, and other compounds. These volatile compounds were mainly derived from fat oxidation, amino acid catabolism, carbohydrate fermentation, microbial activity, and spices (Kaban and Kaya, 2009). The principal component analysis (PCA) of flavor compounds among treatment groups at 28 days of sausage fermentation is shown in Figure 6C. It was found that the control groups, including CK, CK-1, and CK-3, were localized in the third quadrant and clearly scattered from the inoculated groups. Moreover, the inoculated groups of *L. fermentum* YZU-06 (G groups) and mixture starter of *L. fermentum* YZU-06 and *S. saprophyticus* CGMCC 3475 (GQ groups) also showed different distributions of the PCA mapping points. The amounts of volatile compounds in samples inoculated with mixture starters (GQ group 2,863.85 μg/kg, GQ-1 group 3,033.21 μg/kg, GQ-3 group 3,585.83 μg/kg) were higher than those in the control groups (average value of 2,807.53 μg/kg). Thus, it is indicated that the addition of Leu and mixed starter plays an important role in enriching the types and contents of flavor substances in fermented sausage.

The 3-methylbutanal/butanal/butyrate were derived from catabolism of Leu (Beck et al., 2004; Yu et al., 2020), and these compounds were the focus of attention in this study. The addition of 3 mM Leu and mixed-starter culture resulted in higher branched-chain aldehyde/alcohol/acid in the GQ group than that of the other groups (*P* < 0.05). Among them, the content of 3-methylbutanal (17.53 μg/kg) in the GQ-3 group was five times higher than that in the group CK (3.47 μg/kg) and 1.3 times higher than that of the G-3 group. The amount of 3-methylbutanol (5.48 μg/kg) and 2-methylpropanol (1.92 μg/kg) produced by the GQ-3 group was significantly higher than the other groups (*P* < 0.05). Microbial fermentation promoted the conversion of muscle proteins to FAAs in fermented sausages. BCAAs can be converted into α-keto acids by microbial transaminases, which was the first rate-limiting step for the conversion of BCAAs to branched aldehydes. As an important intermediate, α-keto acids can be further transformed into various flavor compounds through non-oxidative decarboxylation or oxidative dehydrogenation (Smit et al., 2004). Aldehydes can be converted into the corresponding alcohols and carboxylic acids by alcohol dehydrogenase and aldehyde dehydrogenase, respectively (Chen et al., 2015). 2-Methylpropanal produced by valine can be oxidized to 2-methylpropionic acid by aldehyde dehydrogenase or reduced to 2-methylpropanol by alcohol dehydrogenase, respectively. Moreover, 2- and 3-methylbutanal produced by Leu and Ile can be oxidized to 2- and 3-methylbutanal by aldehyde dehydrogenase or reduced to 2- and 3-methylbutanol by alcohol dehydrogenase. These compounds produce malt, fruity, and sweaty flavors, which were identified as the key volatile compounds in cured ham and cured loin (Bosse et al., 2021). In addition, the most abundant aldehydes in fermented sausage were non-anal formed by the oxidation of N-9 polyunsaturated fatty acids, which delivered the smell of carnation, citrus, and laurel (Olivares et al., 2011).

Esters are low-odor threshold compounds and give fermented sausage fruit aroma (Sun et al., 2010). Ester was the most abundant in the G group. Ethyl butyrate and methyl 2-methylbutyrate were mostly detected in the inoculated groups and related to the post-esterification of methyl branched-chain by transmination of microbial metabolism of BCAAs (Schmidt and Berger, 1998). Ethyl butyrate in the G-3 group (2,969.18 μg/kg) and methyl 2-methylbutyrate in GQ-3 group (5.23 μg/kg) were significantly higher than that of the other groups (*P* < 0.05). In addition, many esters identified in the present study were ethyl esters, which produced fruity odors (Chen et al., 2017). The high content of ethyl compounds may be due to the decomposition of carbohydrates by *L. fermentum* YZU-06. Sun et al. (2010) showed that ethanol played an important role in the flavor of Cantonese sausage. In the present study, ethanol was identified to have the highest content in alcohols, among which the GQ group had the lowest ethanol content.

Acids have great implications on the flavor development of sausage, including acetic acid and hexanoic acid, which are derived from the degradation of carbohydrates (Liu et al., 2003). The GQ-3 group exhibited significantly higher 3-methylbutanoic acid (9.12 μg/kg) than the other treatment groups (*P* < 0.05). Ketones could be produced mainly through lipid oxidation, microbial fermentation, and carbohydrate catabolism. Two ketones were detected, of which 2-pentanone was produced by microbial β-oxidation in the presence of *Staphylococcus* (Leroy et al., 2006). Hydrocarbons, including four alkanes and five alkenes, were also detected in the volatile flavor substances of sausages, which may be the product of lipid auto-oxidation. However, the presence of alkanes might not be an important contributor to the flavor due to their high threshold value (Latorre-Moratalla et al., 2011). The alkenes, including α-curmene, D-limonene, β-caryophyllene, cedarene, and β-elemene, may be obtained from spices such as onion and ginger powder and spice powder (Montanari et al., 2016).

### 3.7. Sensory analysis

A sensory analysis is essential for meat products to meet consumer demand and recognition (Ruiz-Capillas et al., 2021). As shown in Table 2, no significant difference was found in texture scores among the inoculated groups of *L. fermentum* YZU-06 only and mixed-starter groups (*P* > 0.05), while the color scores in the mixed-starter groups were significantly higher than that of the control groups (*P* < 0.05). The inoculated groups, except the G group, showed significantly higher overall flavor scores than those in the control groups (*P* < 0.05). Two inoculated groups (GQ-1 and GQ-3) presented greater taste scores than those of the control groups (*P* < 0.05). This was attributed to the fact that the inoculation of the starter could promote the hydrolysis of protein and the subsequent release of peptides and FAA for the development of flavor and taste during fermentation (Hu et al., 2022). Taken together, the addition of starter and Leu significantly improved the sensory quality of the sausage.

**TABLE 2:** Scores of sensory evaluation of sausages among different treatments.

Time (d)	Group									SE	*P*-value		
	CK	CK-1	CK-3	G	G-1	G-3	GQ	GQ-1	GQ-3		Strain	BCAAs	Strain*BCAAs
Color	6.23^c^	6.53^c^	6.68^bc^	7.16^ab^	7.28^ab^	7.33^a^	7.50^a^	7.46^a^	7.76^a^	0.04	<0.05	0.22	0.89
Flavor	7.10^b^	7.15^b^	7.18^b^	7.60^ab^	7.83^a^	7.95^a^	8.23^a^	8.00^a^	8.03^a^	0.02	<0.05	0.88	0.66
Texture	7.43^a^	7.35^ab^	7.20^ab^	6.79^c^	7.05^b^	7.43^a^	6.90^c^	7.05^b^	7.54^a^	0.14	0.32	0.10	0.18
Taste	7.25^b^	7.13^c^	7.10^c^	7.15^c^	7.68^ab^	7.70^ab^	7.75^ab^	7.85^a^	7.90^a^	0.07	<0.05	0.54	0.54

*Indicates the interaction between strains and branched-chain amino acids. Different superscript letters (a–c) indicate a significant difference among treatment groups (*P* < 0.05).

## 7. Conclusion

The primary outcome of this study was to develop the fermented sausage by directional production of 3-methylbutanal *via* Leu supplement and the selected culture, *L. fermentum* YZU-06 and *S. saprophyticus* CGMCC 3745. The combination of Leu and strain applied to fermented sausage provides a novel strategy to not only improve the diversity of flavor compounds, but also the overall quality of the fermented sausage. Therefore, *L. fermentum* YZU-06 has great potential as a starter for the production of fermented meat products. The mechanism of flavor development is the interaction between protein, lipids, microorganisms, spices, external environment, and processing parameters and needs to be further studied.

## Data availability statement

The original contributions presented in this study are included in the article/Supplementary material, further inquiries can be directed to the corresponding author.

## Author contributions

HY and RL: conceptualization and resources. YM: methodology, investigation, and writing the original draft preparation. MW: software. RL, HY, and QG: validation. CL: formal analysis, writing, reviewing, and editing. RL: data curation. JX: supervision. HY: funding acquisition. All authors have read and agreed to the published version of the manuscript.
