# Supplementary material for: Effects of the addition of leucine on flavor and quality of sausage fermented by Lactobacillus fermentum YZU-06 and Staphylococcus saprophyticus CGMCC 3475
Source: Front Microbiol. 2023 Feb 2;13:1118907. doi: 10.3389/fmicb.2022.1118907 (PMC9932774; doi:10.3389/fmicb.2022.1118907)
Supplement: Supplementary file 1 [file Table_1.DOCX]

Supplementary Material

**Effects of the addition of leucine on** **flavor** **and quality of sausage fermented by selected starter culture**

**Rui Liu^1^, Yong Ma^1^, Lei Chen^1^, Chenyan Lu^1^, Qingfeng Ge^1^, Mangang Wu^1^, Jun Xi^2^, Hai Yu^1,*^**

*** Correspondence:**Fax (Tel.): +86-0514-87978022
Email: yuhai@yzu.edu.cn (Hai Yu)

**Table S1. Detailed volatile substances of sausages among treatment groups at 28 d of fermentation**

| Flavor substance | Group | | | | | | | | | SE | P-Value | | |
| --- | --- | --- | --- | --- | --- | --- | --- | --- | --- | --- | --- | --- | --- |
|  | CK | CK-1 | CK-3 | G | G-1 | G-3 | GQ | GQ-1 | GQ-3 |  | Strain | Leu | Strain*Leu |
| **Esters** | **1726.13^g^** | **2154.5^e^** | **1699.25^h^** | **3540.9^a^** | **2765.36^c^** | **2969.18^b^** | **2116.1^f^** | **2157.13^e^** | **2636.59^d^** | **15.43** | **<0.05** | **<0.05** | **<0.05** |
| Ethyl 3-methylbutyrate | nd | 0.58^f^ | nd | 5.21^b^ | 1.97^c^ | 7.71^a^ | 0.81^e^ | 1.30^d^ | 5.23^b^ | 0.01 | <0.05 | <0.05 | <0.05 |
| Methyl 2-methylbutyrate | nd | 0.21^e^ | 0.12^f^ | 0.13^f^ | 0.53^b^ | 0.34^d^ | 0.53^b^ | 0.43^c^ | 0.63^a^ | 0.03 | <0.05 | <0.05 | <0.05 |
| [Methyl butyrate](https://pubchem.ncbi.nlm.nih.gov/compound/12180) | 17.25^e^ | 20.83^d^ | 61.48^a^ | 34.22^b^ | 16.60^e^ | 31.64^c^ | 5.61^h^ | 7.46^g^ | 13.13^f^ | 0.91 | <0.05 | <0.05 | <0.05 |
| Ethyl butyrate | 21.63^g^ | 41.93^b^ | 22.63^g^ | 72.61^a^ | 41.63^b^ | 37.20^c^ | 25.22^f^ | 29.60^e^ | 35.11^d^ | 1.09 | <0.05 | <0.05 | <0.05 |
| [Ethyl pentanoate](https://pubchem.ncbi.nlm.nih.gov/compound/10882) | 18.43^d^ | 23.58^c^ | nd | 26.32^b^ | 23.28^c^ | 26.81^b^ | 30.07^a^ | 18.73^d^ | 18.82^d^ | 0.55 | <0.05 | <0.05 | <0.05 |
| [Methyl hexanoate](https://pubchem.ncbi.nlm.nih.gov/compound/7824) | 135.58^i^ | 220.19^g^ | 193.61^h^ | 343.02^c^ | 294.09^d^ | 497.37^a^ | 413.30^b^ | 240.26^f^ | 281.05^e^ | 3.44 | <0.05 | <0.05 | <0.05 |
| [Butyl hexanoate](https://pubchem.ncbi.nlm.nih.gov/compound/12294) | nd | nd | nd | 17.25^a^ | 16.51^b^ | 16.06^c^ | 11.25^f^ | 12.38^e^ | 15.34^d^ | 0.16 | <0.05 | <0.05 | <0.05 |
| [Ethyl caproate](https://pubchem.ncbi.nlm.nih.gov/compound/31265) | 1166.07^g^ | 1480.18^e^ | 1162.55^g^ | 2698.22^a^ | 2029.34^b^ | 2033.75^b^ | 1417.11^f^ | 1577.82^d^ | 1960.58^c^ | 12.33 | <0.05 | <0.05 | <0.05 |
| [Ethyl octanoate](https://pubchem.ncbi.nlm.nih.gov/compound/7799) | 183.15^a^ | 164.02^c^ | 125.35^g^ | 174.73^b^ | 172.01^b^ | 157.95^d^ | 102.74^h^ | 135.01^f^ | 150.35^e^ | 3.19 | <0.05 | <0.05 | <0.05 |
| [Ethyl heptanoate](https://pubchem.ncbi.nlm.nih.gov/compound/7797) | 65.27^d^ | 87.34^a^ | 86.69^a^ | 85.37^a^ | 72.88^c^ | 75.20^b^ | 51.85^f^ | 58.89^e^ | 71.35^c^ | 1.19 | <0.05 | <0.05 | <0.05 |
| [Ethyl decanoate](https://pubchem.ncbi.nlm.nih.gov/compound/8048) | 71.49^a^ | 55.74^b^ | 26.07^h^ | 52.24^c^ | 49.22^d^ | 50.68^cd^ | 32.77^g^ | 42.19^f^ | 46.26^e^ | 1.03 | <0.05 | <0.05 | <0.05 |
| [Methyl decanoate](https://pubchem.ncbi.nlm.nih.gov/compound/8050) | nd | nd | nd | nd | nd | nd | 2.15^b^ | 2.21^b^ | 2.62^a^ | 0.09 | <0.05 | <0.05 | <0.05 |
| [Ethyl nonanoate](https://pubchem.ncbi.nlm.nih.gov/compound/31251) | nd | nd | nd | 8.45^b^ | 9.16^a^ | 9.01^a^ | 6.50^c^ | 6.62^c^ | 8.33^b^ | 0.11 | <0.05 | <0.05 | <0.05 |
| [Ethyl (E)-hept-2-enoate](https://pubchem.ncbi.nlm.nih.gov/compound/5358363) | 22.63^a^ | 16.99^b^ | 14.72^c^ | 9.34^e^ | 10.44^d^ | 10.62^d^ | 6.43^f^ | 6.96^f^ | 9.86^de^ | 0.42 | <0.05 | <0.05 | <0.05 |
| [Ethyl laurate](https://pubchem.ncbi.nlm.nih.gov/compound/7800) | 1.59^f^ | 3.51^ab^ | 2.76^d^ | 3.62^a^ | 3.31^bc^ | 3.25^bc^ | 2.33^e^ | 3.20^c^ | 3.01^cd^ | 0.16 | <0.05 | <0.05 | <0.05 |
| [Ethyl myristate](https://pubchem.ncbi.nlm.nih.gov/compound/31283) | 2.48^e^ | 4.51^a^ | 3.21^d^ | 3.64^c^ | 3.97^b^ | 3.03^d^ | 2.34^f^ | 3.62^c^ | 3.19^d^ | 0.14 | <0.05 | <0.05 | <0.05 |
| Methyl 12-(2-Octylcyclopropyl) | nd | nd | nd | 1.38^b^ | 1.06^c^ | 1.22^b^ | 0.81^d^ | 0.89^d^ | 2.64^a^ | 0.04 | <0.05 | <0.05 | <0.05 |
| [Methyl palmitate](https://pubchem.ncbi.nlm.nih.gov/compound/8181) | nd | nd | nd | nd | 1.35^a^ | nd | 0.83^c^ | 1.35^a^ | 1.13^b^ | 0.02 | <0.05 | <0.05 | <0.05 |
| Ethyl palmitate | nd | 6.89^b^ | nd | nd | 7.39^a^ | 6.04^c^ | 3.60^e^ | 6.75^b^ | 5.28^d^ | 0.12 | <0.05 | <0.05 | <0.05 |
| [Octaethylene glycol monododecyl ether](https://pubchem.ncbi.nlm.nih.gov/compound/123921) | 20.49^a^ | 13.03^b^ | nd | 2.16^c^ | nd | nd | nd | nd | nd | 0.33 | <0.05 | <0.05 | <0.05 |
| [Dibutyl phthalate](https://pubchem.ncbi.nlm.nih.gov/compound/3026) | nd | 13.72^a^ | nd | 1.42^c^ | 10.50^b^ | nd | nd | nd | 1.57^c^ | 0.16 | <0.05 | <0.05 | <0.05 |
| [Ethyl 9-hexadecenoate](https://pubchem.ncbi.nlm.nih.gov/compound/5364759) | nd | 1.18^c^ | nd | 1.50^a^ | nd | 1.22^c^ | nd | 1.37^b^ | 1.01^d^ | 0.15 | <0.05 | <0.05 | <0.05 |
| **Aldehydes** | **50.98^g^** | **64.91^e^** | **90.85^a^** | **68.25^d^** | **81.90^b^** | **81.92^b^** | **58.10^f^** | **72.18^c^** | **91.79^a^** | **1.17** | <0.05 | <0.05 | <0.05 |
| [3-Methylbutanal](https://pubchem.ncbi.nlm.nih.gov/compound/11552) | 3.47^h^ | 3.78^h^ | 5.73^g^ | 10.49^d^ | 9.60^e^ | 13.75^c^ | 6.39^f^ | 15.25^b^ | 17.53^a^ | 0.31 | <0.05 | <0.05 | <0.05 |
| 2-Methylbutanal | 2.85^d^ | 0.45^h^ | 1.56^g^ | 2.85^d^ | 4.58^c^ | 2.17^f^ | 2.61^e^ | 6.96^a^ | 5.91^b^ | 0.10 | <0.05 | <0.05 | <0.05 |
| [2-Methylpropanal](https://pubchem.ncbi.nlm.nih.gov/compound/6561) | 2.96^g^ | 2.52^h^ | 4.42^e^ | 3.49^f^ | 4.12^d^ | 7.74^a^ | 5.92^b^ | 5.76^b^ | 4.87^c^ | 0.13 | <0.05 | <0.05 | <0.05 |
| [2-Heptenal, (Z)](https://pubchem.ncbi.nlm.nih.gov/compound/5362616) | 11.82^f^ | 12.42^e^ | 12.87^d^ | 13.23^cd^ | 14.13^b^ | 13.40^c^ | 11.34^g^ | 13.47^c^ | 21.16^a^ | 0.24 | <0.05 | <0.05 | <0.05 |
| [Nonanal](https://pubchem.ncbi.nlm.nih.gov/compound/31289) | 29.35^f^ | 43.19^b^ | 62.35^a^ | 37.17^d^ | 42.69^b^ | 41.11^c^ | 28.01^g^ | 25.73^h^ | 37.00^d^ | 0.66 | <0.05 | <0.05 | <0.05 |
| [(Z)-2-Decenal](https://pubchem.ncbi.nlm.nih.gov/compound/5354834) | nd | nd | 3.09^a^ | 1.00^f^ | 2.68^b^ | 1.83^d^ | 1.61^e^ | 2.26^c^ | 2.66^b^ | 0.04 | <0.05 | <0.05 | <0.05 |
| [2-Bromooctadecanal](https://pubchem.ncbi.nlm.nih.gov/compound/537255) | 0.51^f^ | nd | 0.78^d^ | nd | 1.36^a^ | nd | 0.71^e^ | 0.93^c^ | 1.05^b^ | 0.03 | <0.05 | <0.05 | <0.05 |
| Octadecanal | nd | 2.52^b^ | nd | nd | 2.70^a^ | 1.90^c^ | 1.48^e^ | 1.80^d^ | 1.57^e^ | 0.05 | <0.05 | <0.05 | <0.05 |
| **Alcohols** | **228.62^ef^** | **271.73^c^** | **284.56^b^** | **328.44^a^** | **225.41^f^** | **246.48^d^** | **149.39^h^** | **181.75^g^** | **233.89^e^** | **3.5** | <0.05 | <0.05 | <0.05 |
| [3-Methylbutanol](https://pubchem.ncbi.nlm.nih.gov/compound/31260) | 0.14^h^ | 0.43^g^ | 1.67^e^ | 2.53^d^ | 3.58^c^ | 3.70^c^ | 0.85^f^ | 4.28^b^ | 5.48^a^ | 0.1 | <0.05 | <0.05 | <0.05 |
| [2-Methylbutanol](https://pubchem.ncbi.nlm.nih.gov/compound/8723) | 0.55^ef^ | 0.43^f^ | 0.67^e^ | 1.37^d^ | 1.56^c^ | 1.61^c^ | 1.34^d^ | 2.84^a^ | 2.36^b^ | 0.07 | <0.05 | <0.05 | <0.05 |
| [2-Methylpropanol](https://pubchem.ncbi.nlm.nih.gov/compound/6560) | nd | nd | 0.33^e^ | 0.52^d^ | 1.38^b^ | 0.46^d^ | 1.08^c^ | 1.43^b^ | 1.92^a^ | 0.05 | <0.05 | <0.05 | <0.05 |
| [Ethanol](https://pubchem.ncbi.nlm.nih.gov/compound/702) | 152.87^d^ | 170.96^c^ | 190.17^b^ | 211.86^a^ | 106.80^g^ | 123.46^e^ | 74.47^i^ | 93.01^h^ | 116.97^f^ | 2.85 | <0.05 | <0.05 | <0.05 |
| [1-Hexanol](https://pubchem.ncbi.nlm.nih.gov/compound/8103) | 30.14^g^ | 45.71^d^ | 21.32^h^ | 53.24^b^ | 51.76^c^ | 54.83^a^ | 42.37^e^ | 35.09^f^ | 46.50^d^ | 0.83 | <0.05 | <0.05 | <0.05 |
| [Linalool](https://pubchem.ncbi.nlm.nih.gov/compound/6549) | 44.90^a^ | 30.74^c^ | 40.88^b^ | 27.70^c^ | 27.89^c^ | 30.12^c^ | 15.91^g^ | 17.92^f^ | 21.55^e^ | 0.52 | <0.05 | <0.05 | <0.05 |
| [Terpinen-4-ol](https://pubchem.ncbi.nlm.nih.gov/compound/11230) | nd | 10.45^d^ | 12.28^b^ | 11.21^d^ | 11.617^c^ | 12.75^a^ | 4.38^f^ | 8.34^e^ | 10.38^d^ | 0.19 | <0.05 | <0.05 | <0.05 |
| [Tert-hexadecyl mercaptan](https://pubchem.ncbi.nlm.nih.gov/compound/545889) | nd | 4.27^b^ | 2.7^d^ | 1.49^f^ | 7.39^a^ | 1.24^g^ | 2.04^e^ | 4.02^c^ | 1.12^g^ | 0.08 | <0.05 | <0.05 | <0.05 |
| [(Z)-Hept-2-en-1-ol](https://pubchem.ncbi.nlm.nih.gov/compound/5364958) | nd | nd | nd | nd | nd | 1.31^d^ | 1.57^c^ | 2.36^b^ | 12.02^a^ | 0.09 | <0.05 | <0.05 | <0.05 |
| [2-Methylhexadecan-1-ol](https://pubchem.ncbi.nlm.nih.gov/compound/17218) | nd | 8.70^c^ | 14.49^a^ | 11.12^b^ | 4.69^e^ | 3.13^f^ | 2.09^g^ | 6.64^d^ | 10.79^b^ | 0.22 | <0.05 | <0.05 | <0.05 |
| Cinnamyl alcohol | nd | nd | nd | 7.35^c^ | 8.71^b^ | 13.81^a^ | 3.26^f^ | 5.77^d^ | 4.74^e^ | 0.08 | <0.05 | <0.05 | <0.05 |
| **Acids** | **13.26^f^** | **7.86^g^** | **6.27^h^** | **20.86^c^** | **15.12^d^** | **20.49^c^** | **13.92^f^** | **24.35^b^** | **29.29^a^** | **0.39** | <0.05 | <0.05 | <0.05 |
| [3-Methylbutanoic acid](https://pubchem.ncbi.nlm.nih.gov/compound/10430) | 0.24^g^ | 2.49^f^ | 4.24e | 5.27^d^ | 4.36^e^ | 6.50^c^ | 4.30^e^ | 8.34^b^ | 9.12^a^ | 0.14 | <0.05 | <0.05 | <0.05 |
| [2-Methylbutanoic acid](https://pubchem.ncbi.nlm.nih.gov/compound/8314) | 1.20^ef^ | 1.02^f^ | 1.37^e^ | 3.51^a^ | 2.44^bc^ | 2.10^d^ | 1.38^e^ | 2.22^cd^ | 2.54^b^ | 0.16 | <0.05 | <0.05 | <0.05 |
| [2-Methylpropanoic acid](https://pubchem.ncbi.nlm.nih.gov/compound/6590) | nd | 0.22^d^ | 0.28^d^ | 0.87^c^ | 1.38^a^ | 1.13^b^ | 0.96^c^ | 1.41^a^ | 1.15^b^ | 0.08 | <0.05 | <0.05 | <0.05 |
| [Acetic acid](https://pubchem.ncbi.nlm.nih.gov/compound/176) | nd | nd | nd | 5.38^d^ | 5.021^e^ | 7.56^c^ | 2.33^f^ | 8.51^b^ | 10.52^a^ | 0.10 | <0.05 | <0.05 | <0.05 |
| [Hexanoic acid](https://pubchem.ncbi.nlm.nih.gov/compound/8892) | 0.42^fg^ | 0.51^f^ | 0.37^g^ | 1.38^d^ | 0.91^e^ | 0.26^h^ | 2.08^b^ | 2.52^a^ | 1.65^c^ | 0.05 | <0.05 | <0.05 | <0.05 |
| [9-Hexadecenoic acid](https://pubchem.ncbi.nlm.nih.gov/compound/4668) | 9.25^a^ | 1.82^b^ | nd | nd | nd | nd | nd | nd | nd | 0.05 | <0.05 | <0.05 | <0.05 |
| [Palmitic acid](https://pubchem.ncbi.nlm.nih.gov/compound/985) | 2.13^a^ | 1.78^b^ | nd | nd | nd | nd | nd | nd | nd | 0.03 | <0.05 | <0.05 | <0.05 |
| [3-Hydroxydodecanoic acid](https://pubchem.ncbi.nlm.nih.gov/compound/94216) | nd | nd | nd | 4.42^a^ | 0.99^e^ | 2.92^c^ | 2.85^c^ | 1.34^d^ | 4.28^b^ | 0.07 | <0.05 | <0.05 | <0.05 |
| **Alkanes** | **11.59^g^** | **28.62^c^** | **46.94^b^** | **13.37^e^** | **18.28^d^** | **56.02^a^** | **12.50^f^** | **11.54^g^** | **14.26^e^** | **0.49** | <0.05 | <0.05 | <0.05 |
| [2,6-Dimethyldecane](https://pubchem.ncbi.nlm.nih.gov/compound/139395) | nd | 2.46^f^ | 8.31^b^ | 3.73^e^ | 5.32^d^ | 12.84^a^ | 6.04^c^ | 1.79^g^ | nd | 0.26 | <0.05 | <0.05 | <0.05 |
| [Dodecane](https://pubchem.ncbi.nlm.nih.gov/compound/8182) | 11.59^d^ | 14.38^c^ | 25.88^b^ | 9.64^g^ | nd | 27.85^a^ | 6.45^h^ | 5.72^i^ | 11.05^f^ | 0.04 | <0.05 | <0.05 | <0.05 |
| 6-Methyltridecane | nd | 4.27^b^ | 5.12^a^ | nd | nd | nd | nd | nd | nd | 0.04 | <0.05 | <0.05 | <0.05 |
| [2,3-Dimethyldecane](https://pubchem.ncbi.nlm.nih.gov/compound/86544) | nd | 7.50^c^ | 7.62^c^ | nd | 12.95^b^ | 15.33^a^ | nd | 4.02d | 3.21^e^ | 0.17 | <0.05 | <0.05 | <0.05 |
| **Alkenes** | **118.54^g^** | **161.86^c^** | **202.05^a^** | **134.23^f^** | **173.02^b^** | **141.88^e^** | **94.80^h^** | **131.79^f^** | **146.57^d^** | **2.14** | <0.05 | <0.05 | <0.05 |
| [α-Curcumene](https://pubchem.ncbi.nlm.nih.gov/compound/92139) | 118.54^b^ | 95.29^e^ | 109.27^d^ | 119.67^b^ | 143.23^a^ | 113.37^c^ | 74.26^f^ | 107.66^d^ | 113.55^c^ | 1.43 | <0.05 | <0.05 | <0.05 |
| [D-limonene](https://pubchem.ncbi.nlm.nih.gov/compound/440917) | nd | 54.08^b^ | 69.96^a^ | nd | nd | nd | nd | nd | nd | 0.47 | <0.05 | <0.05 | <0.05 |
| [β-Caryophyllene](https://pubchem.ncbi.nlm.nih.gov/compound/5281515) | nd | 6.18^cd^ | 6.54^c^ | 7.93^a^ | 7.49^b^ | 7.29^b^ | 3.77^e^ | 5.89^d^ | 6.35^c^ | 0.24 | <0.05 | <0.05 | <0.05 |
| [Cedarene](https://pubchem.ncbi.nlm.nih.gov/compound/6431015) | nd | 6.29^c^ | 7.14^a^ | 6.62^b^ | nd | nd | 3.67^d^ | nd | 6.38^c^ | 0.13 | <0.05 | <0.05 | <0.05 |
| [β-Elemene](https://pubchem.ncbi.nlm.nih.gov/compound/6918391) | nd | nd | 9.12^e^ | nd | 22.29^a^ | 21.21^b^ | 13.08^d^ | 18.23^c^ | 20.27^b^ | 0.59 | <0.05 | <0.05 | <0.05 |
| **Ketones** | **40.22^c^** | **49.45^a^** | **4.69^g^** | **41.69^bc^** | **18.71^f^** | **41.08^c^** | **26.88^e^** | **36.46^d^** | **43.18^b^** | **0.87** | <0.05 | <0.05 | <0.05 |
| [2-Pentanone](https://pubchem.ncbi.nlm.nih.gov/compound/7895) | 40.22^b^ | 49.45^a^ | nd | 37.99^c^ | 13.97^f^ | 37.36^c^ | 24.47^e^ | 33.29^d^ | 39.50^b^ | 0.83 | <0.05 | <0.05 | <0.05 |
| [2-Undecanone](https://pubchem.ncbi.nlm.nih.gov/compound/8163) | nd | nd | 4.69^a^ | 3.69^b^ | 4.73^a^ | 3.72^b^ | 2.41^d^ | 3.16^c^ | 3.68^b^ | 0.08 | <0.05 | <0.05 | <0.05 |
| **Others** | **253.22^g^** | **390.90^f^** | **515.48^b^** | **476.34^d^** | **566.04^a^** | **498.22^c^** | **392.13^f^** | **417.97^e^** | **390.23^f^** | **2.12** | <0.05 | <0.05 | <0.05 |
| (Z)-Anethole | 253.22^g^ | 390.90^f^ | 451.84^d^ | 476.34^c^ | 566.04^a^ | 498.22^b^ | 392.13^f^ | 417.97^e^ | 390.23^f^ | 2.08 | <0.05 | <0.05 | <0.05 |
| 4-Allylanisole | nd | nd | 63.63 | nd | nd | nd | nd | nd | nd | 0.19 | <0.05 | <0.05 | <0.05 |
| **Total** | **2442.60^h^** | **3129.87^e^** | **2850.12^g^** | **4624.12^a^** | **3863.87^c^** | **4055.31^b^** | **2863.85^g^** | **3033.21^f^** | **3585.83^d^** | **17.90** | <0.05 | <0.05 | <0.05 |

Note: “nd” means no detection. Different lowercase letters (a-i) means significant difference between different treatment groups of the same substance (*P* < 0.05).
